# School-Based Alcohol and Tobacco Prevention Strategies: A Scoping Review and the Missing Role of School Nurses

**DOI:** 10.3390/children13040453

**Published:** 2026-03-26

**Authors:** Paula Concha-Gacitua, Amalia Sillero Sillero, Sonia Ayuso-Margañon, Maria J. Golusda, Ana Maria Montserrat-Gala, Eva Gutiérrez-Naharro, Raquel Ayuso-Margañon

**Affiliations:** 1 Faculty of Medicine, Universidad del Desarrollo, Las Condes 7610658, Santiago, Chile; p.concha@udd.cl (P.C.-G.); mjesus.golusda@udd.cl (M.J.G.); 2 Escola Universitària Gimbernat, Adscrita a la Universitat Autònoma de Barcelona (UAB), 08174 Sant Cugat, Spain; egutinaharro@gmail.com; 3 Public Health Nursing, Mental Health and Maternal-Child Health, Faculty of Nursing, University of Barcelona, 08907 L’Hospitalet de Llobregat, Spain; soniaayuso@ub.edu.es; 4 North Florida Primary Health Care, Florida Nord, L’Hospitalet de Llobregat, 08905 Barcelona, Spain; 5 Department of Medicine, Autonomous University of Barcelona (UAB), 08193 Barcelona, Spain; anamaria.montserrat@uab.cat; 6 Social Determinants and Health Education Research Group (SDHEd), Hospital del Mar Research Institute, 08003 Barcelona, Spain; rayuso@esimar.edu.es; 7 Hospital del Mar Nursing School (ESIHMar), Universitat Pompeu Fabra, 08003 Barcelona, Spain

**Keywords:** adolescent health, substance use prevention, health education, school health service, alcohol drinking, tobacco use

## Abstract

**Highlights:**

**What are the main findings?**

- School-based programmes to prevent alcohol and tobacco use are diverse and increasingly digital, but they continue to face challenges in engagement, fidelity and long-term adherence.
- None of the reviewed interventions involved school nurses, despite their recognised role in adolescent health promotion

**What are the implications of the main findings?**

- Strengthening collaboration among teachers, families, and health professionals could enhance continuity, early detection, and overall programme quality.
- Digital and hybrid approaches require relational support, structured monitoring, and clear implementation roles; combining online tools with face-to-face interaction may improve engagement, family involvement, and long-term sustainability.

**Abstract:**

**Background/Objectives**: Alcohol and tobacco use in adolescence are major public health concerns that shape long-term health trajectories and undermine healthy behaviour development. Schools are key settings for health promotion, offering structured environments to foster self-regulation, social skills, and protective behaviours. This scoping review mapped recent school-based educational strategies designed to prevent alcohol and tobacco use among adolescents and to examine whether the included studies reported any involvement of school nurses. **Methods:** Review followed Arksey and O’Malley’s framework and adhered to JBI guidance and PRISMA-ScR. Searches were conducted in PubMed and Web of Science (2019–2024) to identify school-based educational interventions targeting alcohol and/or tobacco use among primary or secondary school children. The primary search targeted prevention strategies, complemented by nursing-related terms to identify nurse involvement. A standardised charting form captured study characteristics, intervention formats, theoretical foundations, implementation factors, and any reported participation of health professionals. Data extraction was performed independently by two reviewers. **Results**: Eleven studies met the inclusion criteria. Most were randomised controlled trials (81.8%). Educational strategies included online (45.5%), hybrid (27.3%), and face-to-face (27.3%) formats. Programs focused on social skills, self-regulation, harm reduction, or resilience. Digital formats were cost-effective but showed challenges in engagement and sustained participation, while face-to-face or hybrid approaches offered relational support but were vulnerable to implementation drift. No study reported nurse involvement. **Conclusions:** School-based prevention strategies can contribute to healthier behaviours related to substance use by reinforcing socioemotional competencies and reducing early exposure to substances. However, persistent barriers such as low engagement, inconsistent delivery, and the absence of health professionals limit their impact. The role of school nurses could be considered in future school-based prevention programmes.

## 1. Introduction

Alcohol and tobacco use among school-aged children remains a global public health problem with significant consequences for physical, cognitive and social development. Globally, about 25% of youths have tried alcohol and 10% use tobacco [1,2]. In Europe, more than 57% of 15-year-olds have tried alcohol, and almost 40% have consumed it in the last 30 days, while the use of e-cigarettes reaches 20% in this age group [3]. These behaviours, starting in a vulnerable stage, affect brain development, memory, learning, and increase risks of accidents, violence, school failure and dependence [4,5]. Tobacco use, for its part, promotes nicotine addiction and increases the likelihood of chronic diseases such as cancer and cardiovascular disease [6]. The global burden attributable to these behaviours remains high, with millions of years of life lost to disability and premature death in school-aged children [7].

Particularly between the ages of 12 and 18, young people are most likely to initiate and escalate their use of alcohol and tobacco [8]. This period is marked by heightened vulnerability to risk-taking, stronger peer influence and the transition into secondary education, developmental and contextual factors (such as school environment, family dynamics and community influences) consistently linked to the onset of substance use [9].

Schools are strategic for interventions that promote social skills, self-regulation and healthy decision-making [8,9]. Schools reach most adolescents, integrate content and coordinate with families and health services [10,11]. For this reason, international organisations such as the World Health Organisation (WHO) and the European Union have highlighted the importance of incorporating substance use prevention into school health and education frameworks [12,13]. This aligns with the Sustainable Development Goals (SDGs), especially SDG 3 (Health) and SDG 4 (Education), promoting integrated services to reduce inequalities [14,15].

Global strategies such as the Global Action Plan on Alcohol 2022–2030 and the Global Strategy to Accelerate Tobacco Control emphasise prevention, but do not explicitly address school programmes [16]. At the national level, frameworks vary: the Centres for Disease Control and Prevention (CDC) in the United States promotes screening and taxation policies on alcohol but omits specific interventions in schools [17]; the European Union (EU) Drugs Strategy 2021–2030 provides a broad framework on substance use, while Spain’s National Strategy on Addictions includes school activities [18] and Latin America, Chile’s Prevention Programme in Educational Establishments (PPEE) stands out as an example of institutional commitment [19]. These examples reflect progress in policy development; however, significant challenges remain in translating these frameworks into effective, consistent, and sustainable school-based interventions [20].

Despite regulatory advances and growing interest in strengthening health education in schools, there remains considerable variability in how alcohol and tobacco use prevention programmes are designed and implemented [21]. Although regulatory frameworks emphasise the importance of prevention in schools, the available evidence does not specify which educational strategies are implemented or how these recommendations are translated into concrete actions within schools [21,22]. This lack of clarity makes it difficult to understand the real scope of current interventions. It limits the ability to guide evidence-based policies and practices and to identify which approaches are most effective in achieving meaningful outcomes [23].

In addition, the integration of health professionals into school-based programmes remains limited. Nurses, in particular, play a central role in health promotion and are especially trained to address risk behaviours through educational and preventive approaches. Nurses play a key role as a bridge between the education and health systems, contributing skills in prevention, early detection and health education that complement the work of teachers [24,25]. Their participation promotes continuity of care, adaptation of content to real needs, and implementation of evidence-based strategies [26]. International frameworks, such as the WHO’s global standards for health-promoting schools, recommend integrating health professionals into educational programmes to address risk behaviours and promote healthy environments [27]. However, their participation in school programmes for the prevention of alcohol and tobacco use is not clearly documented.

Although the contribution of nurses is recognised in broader school-health promotion frameworks, few studies have examined their involvement in the design, implementation or evaluation of school-based alcohol and tobacco prevention programmes [28]. This gap, together with structural barriers (e.g., limited staffing, time constraints, rigid school schedules and insufficient institutional support) that often limit the integration of health expertise in schools, highlights the need for a synthesis of existing initiatives. A clearer understanding of current educational strategies, and whether any involvement of school nurses is reported within them, is essential for informing future programme development and implementation.

Therefore, the aim of this scoping review was to map recent school-based educational strategies designed to prevent alcohol and tobacco use among adolescents and to examine whether the included studies reported any involvement of school nurses.

## 2. Materials and Methods

### 2.1. Study Design

This scoping review was conducted following the methodological framework proposed by Arksey and O’Malley [29], which is widely recognised for mapping heterogeneous evidence. This approach was appropriate given the variability in school-based prevention strategies and the need to identify gaps rather than assess intervention effectiveness.

The framework facilitated the identification of diverse strategies, including digital, hybrid, and face-to-face formats, and enabled the exploration of implementation challenges. Although the main focus was on educational strategies, the review also examined whether nurses or other health professionals were involved in the design, delivery, or evaluation of interventions. This was considered an exploratory dimension to assess gaps in interprofessional collaboration and inform future policy recommendations.

The review protocol was developed before data collection to ensure methodological transparency and reproducibility. The review followed the Joanna Briggs Institute (JBI) and PRISMA-ScR (Preferred Reporting Items for Systematic Reviews and Meta-Analyses Extension for Scoping Reviews) checklists to ensure methodological rigour [30,31]. The complete checklist is provided as Supplementary Material S1 (Table S1).

### 2.2. Eligibility Criteria

Studies were eligible if they described school-based interventions aimed at preventing alcohol and/or tobacco use among school-aged children. The age range of 12–18 years was selected because it corresponds to the period in which the initiation and escalation of alcohol and tobacco use most commonly occur [8]. In addition, studies frequently report outcomes using broad adolescent categories that overlap with secondary school ages [12]. Focusing on individuals aged 12–18 years, therefore, ensures alignment with both developmental evidence and the structure of school-based interventions, while capturing the population most targeted by prevention efforts.

We included peer-reviewed articles published between January 2019 and December 2024 in English or Spanish. This five-year window was selected to ensure the evidence is current and relevant, reflecting recent technological innovations and policy developments in school health promotion. In particular, the last decade has seen a rapid expansion of digital and hybrid formats, as well as global initiatives such as the WHO Global Alcohol Action Plan and the Sustainable Development Goals, which emphasise integrated approaches to adolescent health [16]. Extending beyond this period could have introduced outdated strategies less aligned with current educational and health frameworks.

No restrictions were applied regarding study design, as scoping reviews aim to capture the breadth of available evidence rather than assess effectiveness.

### 2.3. Search Strategy

Searches were conducted in PubMed and Web of Science. These databases provide comprehensive coverage of health and education literature. While additional databases could have been included, the decision to focus on these two was based on their strong indexing of both health and education research, which aligns with the topic’s interdisciplinary nature.

Grey literature was consulted through reputable health and education organisations (e.g., National Association of School Nurses—NASN, the Spanish Association of Primary Care Paediatrics—AEPAP, and the International Association of School Nurses & Health Promotion—ISNA) to identify guidelines and reports that could inform the mapping of current practices. However, these sources were not systematically integrated into the analysis because they did not meet the eligibility criteria for empirical studies. This decision was made to maintain methodological consistency and focus on peer-reviewed evidence.

The search strategy was developed iteratively by the review team to align with the review’s objectives: mapping school-based educational strategies for preventing alcohol and tobacco use and exploring whether school nurses were involved in these interventions. Keywords were mapped to controlled vocabulary (MeSH terms) in PubMed to maximise sensitivity and specificity, while equivalent controlled terms were applied in Web of Science to ensure precision. The core concepts were derived from the PCC framework (Population, Concept, Context) [29]:

- Population: children and adolescents enrolled in school settings.
- Concept: educational interventions aimed at preventing alcohol and tobacco use.
- Context: school-based programs implemented in primary or secondary education environments.

The search strategy included adolescent-related terms, as this terminology is routinely used in studies involving individuals aged 12–18 years. Additional nursing-related terms were tested to capture any reference to professional involvement. However, when combined with the main concepts, these terms yielded zero results in PubMed and very few in Web of Science. To avoid excessive restriction, the primary search strategy focused on educational interventions without nursing terms. In parallel, a complementary search using nursing-related terms was conducted separately in both databases to explore conceptual references. In PubMed, this search retrieved two papers mentioning school nurses, and in Web of Science, six papers were found. All were excluded because they did not describe educational strategies integrated into the school curriculum: some focused on sexual health, others on clinical services or contextual challenges, and none evaluated interventions for alcohol or tobacco prevention.

The nursing-related component was therefore treated as an exploratory element of the review rather than as a core part of the primary search strategy. Its purpose was to determine whether existing school-based alcohol and tobacco prevention interventions involved school nurses and to highlight potential gaps in the literature. This approach aligns with the scoping review’s aim of mapping available evidence and identifying areas where research is limited or absent.

Although additional databases could have expanded search coverage, PubMed and Web of Science were selected because they index extensive health and education literature, aligning with the aims of the review. This approach is consistent with the methodological flexibility of scoping reviews, which prioritise comprehensiveness within the scope of the research question rather than exhaustive searching [29].

The final search was conducted between February and May 2024. Detailed strategies for each database are provided in Supplementary Material S2 (Table S2). The screening process followed a structured flow: initial database retrieval, title and abstract screening, and full-text assessment against predefined inclusion and exclusion criteria. Two reviewers independently conducted the screening, with discrepancies resolved through discussion or consultation with a third reviewer. This process ensured methodological transparency and minimised selection bias [30].

### 2.4. Quality Assessment

No critical appraisal or risk-of-bias assessment was performed, as this process does not align with the primary purpose of a scoping review. This review aimed to map the scope, and characteristics of the available evidence, identify gaps in the literature, and inform future research [31]. Article selection followed pre-defined eligibility criteria, and the included studies were classified according to the Joanna Briggs Institute levels of evidence for effectiveness [32]. Most included studies (81.8%) were classified as Level 1 evidence, followed by 9.1% at Level 2 and 9.1% at Level 3 (see Table 1 for details). This classification was used for descriptive purposes only and did not inform the interpretation or weighting of the findings, in accordance with the aims of a scoping review [29].

### 2.5. Data Extraction

Following the search strategy, 427 records were initially retrieved from PubMed and Web of Science. After removing duplicates and applying filters, titles and abstracts were screened to exclude studies outside the publication period, those that did not address school-based educational strategies for alcohol and tobacco prevention, and those that did not meet the predefined inclusion criteria. Most exclusions were due to a lack of educational strategies, wrong target substances, or interventions outside school settings. Complementary searches using nursing-related terms in PubMed (*n* = 2) and Web of Science (*n* = 6) were screened but excluded because they did not meet the inclusion criteria.

Eleven studies were retained for full-text review and data extraction. All citations were systematically organised using Mendeley Cite©2022 and imported into Rayyan©2022 to facilitate duplicate removal and screening.

Data extraction was guided by the review objectives and structured to align with the synthesis presented in Table 1. A standardised charting form was developed to ensure consistency and completeness. For each included study, the following variables were collected:

- Reference: bibliographic details (author, year) for traceability.
- Design and level of evidence (JBI): to classify methodological rigour and heterogeneity [32].
- Sample data: country, population characteristics, and age range to contextualise interventions.
- Educational strategies: type of approach, duration, and theoretical framework to map formats and pedagogical models.
- Nursing role: explicit mention or absence of school nurses or other health professionals, as an exploratory dimension to assess gaps in interprofessional collaboration.
- Perceived benefits: reported advantages such as feasibility, cost-effectiveness, or adaptability.
- Perceived limitations: barriers related to engagement, fidelity, or evaluation tools.

These categories were selected to capture the breadth of evidence on formats, outcomes, and implementation challenges, while addressing the specific gap regarding nurse involvement. Data extraction was performed independently by two reviewers, with discrepancies resolved through discussion to minimise bias and ensure reliability.

### 2.6. Data Analysis

Data analysis was primarily descriptive and aligned with the objectives of a scoping review [33]. The extracted information was organised in Microsoft Excel and analysed using SPSS (version 26.0; IBM Corp., Armonk, NY, USA) to generate basic descriptive statistics, including frequencies and proportions. No formal risk-of-bias assessment was conducted, as the aim was to map the breadth of available evidence rather than evaluate intervention effectiveness.

The synthesis focused on mapping key characteristics of the interventions, including their strategies, delivery formats, and reported challenges, and on identifying gaps in the existing evidence. Results were summarised narratively and presented in tables to facilitate comparison across studies.

**Table 1:** The main results of the studies included.

Reference	Design and Level of Evidence JBI *	Sample Data	Educational Strategies	Nursing Role	Perceived Benefits	Perceived Limitations
Debenham et al., 2024 [34]	Randomised Controlled Trials. Level 1. a	Australia. Students aged 15–19 from 8 schools.	The Illicit Project is an online neuroscience-based intervention. It aims to teach basic neuroscience concepts, strategies to reduce the harm caused by substance abuse (including tobacco and alcohol) and promote self-help. The program is delivered through strengths-based learning, using interactive activities such as case studies.	N/A	In an online intervention, it is ensured that the basic components are implemented. In addition, the intervention not only shows results in reducing substance use, but also in attitudes such as binge drinking and increased knowledge about drugs.	Lack of evaluation by scoring the fidelity of teachers participating as guides in the strategy, which could modify and/or alter the implementation of the program in schools.
Slade et al., 2023 [35]	Randomised Controlled Trials. Level 1. a	Australia. School children aged 12–14 and their parents from 12 schools.	EHealth Universal Program Climate Schools Plus (CSP) for parents and students is an online preventive program that provides information on substance use (alcohol and cannabis) and self-management skills development. For students, the web is accessed in class (a cartoon story is displayed, and then a face-to-face group activity is worked on). For parents, the program provides online activities, such as seminars and modules to complete.	N/A	As an online program, it offers accessibility and flexibility.	Low parental participation was observed. The authors attribute this to the fact that online programs generally decrease participation and engagement compared to face-to-face programs.
Griffin et al., 2022 [36]	Randomised Controlled Trials. Level 1. a	USA. Students aged 11–14 from 23 schools.	The hybrid digital intervention is a hybrid program aimed at school children for the prevention of substance abuse (including tobacco and alcohol). Students had to review online modules with content on drugs and then participate in face-to-face sessions (discussions) to address the content of the videos and develop social skills.	N/A	The methodology enables the delivery of sessions and modules in a standardised, efficient timeframe, allowing time for face-to-face activities and enhancing interactions between teachers and students. It is easy and economical to implement, as it requires only a few resources (internet access and a mobile device).	Class time planning is required for face-to-face modules, and trained teachers must comply with the session plan. It has been observed that some teachers do not adhere to the scheduled sessions, which can affect the outcome of the intervention.
Haug et al., 2022 [37]	Randomised Controlled Trials. Level 1. a	Switzerland. Students with an average age of 17.3 years.	Ready4life is a mobile application that aims to promote social and regulatory skills for the reduction of risky behaviours such as alcohol and tobacco use. The App offers interactive features such as quizzes, competitions, and personalised conversations for each user.	N/A	Implementation is easy, inexpensive and can reach a wide range of school children.	They report that participation in the various implementation activities was generally low, attributing this to poor monitoring and follow-up of the online programs.
Layland et al., 2022 [38]	Randomised Controlled Trials. Level 1.c	South Africa. Students from 34 schools with an average age of 14.1 years.	HealthWise is a strategy that teaches self-regulation skills, emotional control, decision-making and healthy attitudes. In this study, teachers’ participation in implementation is included. It consists of 12 face-to-face sessions and uses strategies such as role-play, self-reflection, and knowledge-delivery sessions on tobacco and alcohol use.	N/A	It suggests that a methodology involving teachers can achieve better results in reducing substance use.	The authors state that teachers can modify the sessions, methodology and/or content as they see fit.
Paz Castro et al., 2022 [39]	Randomised Controlled Trials. Level 1.c	Switzerland. Students aged 14 to 17.	SmartCoach is a strategy that uses online feedback and personalised text messages via mobile phones for 22 weeks. The content was based on social cognitive theory and addressed self-management, social and substance use (including tobacco and alcohol) resistance skills.	N/A	It is an easy (automated) and cost-effective strategy to implement.	They report that the effectiveness of digital strategies on substance use prevention fades in the long term.
Ho et al., 2021 [40]	Randomised Controlled Trials. Level 1. a	China. Students aged 12–15 from 30 schools.	An internet quiz game intervention is an online game that asks participants to answer 1000 questions about alcohol consumption. The questions yielded accurate data on alcohol consumption, suggesting that a new peripheral line of thinking can be created by providing concrete data and information.	N/A	Its implementation requires a low budget and achieves positive results in the short and medium term (1 and 3 months).	It reports difficulties with continuity and participation in the game by the population.
Regina Wojcieszek et al., 2021 [41]	Pre-test—post-test. Level 2.d	Poland. School children aged 12–13 in sixth grade.	The “Debate” prevention program is a preventive intervention in which a debate is held (in groups of students) led by one or two teachers, to promote pro-abstinence attitudes and delay the onset of alcohol consumption.	N/A	As an educational activity lasting 3 h, it is easy to incorporate into school activities. It encourages group work among peers.	The authors suggest revising the program’s evaluation instrument, as some questions may lend themselves to personal interpretation.
Martínez-Montilla et al., 2020 [42]	Randomised Controlled Trials. Level 1. a	Spain. Students aged 15–19 from 15 schools.	Alcohol Alert is an online strategy featuring interactive stories about alcohol use. Students choose characters and make decisions, receiving personalised feedback informed by the I-Change model, which focuses on attitudes, social influences, and self-efficacy.	N/A	It is one of the few school-based prevention strategies using computer-tailored interventions and the I-Change model, with proven cost-effectiveness and impact.	Students reported that some messages were too long and boring to read. Additionally, dropout rates exceeded 50%, possibly due to the lack of attendance control in the online format.
de Visser et al., 2020 [43]	Case-controlled study. Level 3.d	United Kingdom. Secondary school students aged 14–16.	Resilience-based alcohol education is an intervention to prevent alcohol use through the development of behavioural skills and resilience based on the IMB model. The program is delivered through face-to-face lessons, supported by videos that reinforce the benefits of maintaining control over alcohol consumption.	N/A	By using the IMB model (information, motivation, and skills) throughout the planning and execution of the program, it can be adapted to the realities and needs of each educational establishment and is flexible to implement.	As a strategy to be implemented by teachers, it can lead to difficulties in the fidelity of the activities. It was reported that some teachers felt uncomfortable conducting the sessions due to the proximity to the students and the embarrassment and shame they felt when discussing these topics with them.
Teesson et al., 2020 [44]	Randomised Controlled Trials. Level 1. a	Australia. School children aged 13–14 from 71 schools.	The Climate Schools-Combined program is an online combined prevention program on substance use (alcohol and cannabis), depression and anxiety. The program covers these three topics together over 12 40-min lessons. The lessons are delivered in the classroom, and the activities were carried out online through cartoon stories (about substance use).	N/A	It is an easy and cost-effective program to implement. In addition, it can prevent the onset of substance use-related diseases at school.	No limitations of the education strategy are reported. The authors recommend extending the follow-up time in this study, given the older age at which alcohol consumption is more pronounced.

Note 1: N/A = No applicable; * Design and level of evidence: JBI levels of evidence were used to classify study designs according to methodological rigour, following the Joanna Briggs Institute guidelines [32].

## 3. Results

### 3.1. Search Outcome

The initial search strategy yielded 407 publications from PubMed and Web of Science, plus 8 articles from complementary searches using nursing-related terms, for a total of 415 articles. After excluding duplicate articles and those that did not meet the study designs described in the inclusion criteria, 271 articles were selected. Subsequently, they were filtered by title, resulting in 79 articles, and then by abstract, leaving 50 publications. During the full-text review, six articles were classified as “reports not retrieved”, leaving a total of 44 articles to review.

After applying the inclusion and exclusion criteria in the full-text review, 33 articles were excluded for the following reasons: language other than English or Spanish (n = 2), duplicates (n = 2), lack of educational strategies (n = 8), did not assess alcohol and/or tobacco use as a target (n = 9), educational strategies conducted outside the educational establishment (n = 2), targeted at university students (n = 3), incorrect drug (n = 1) and ongoing protocols (n = 6). Three of these were initially considered for inclusion but were ultimately excluded because they assessed self-concept knowledge and skills related to alcohol and tobacco use post educational intervention (n = 2) or exclusively analysed e-cigarette use (n = 1). In addition, seven grey literature documents were screened but excluded due to year of publication (n = 3) or study design (n = 4).

**Figure 1:**
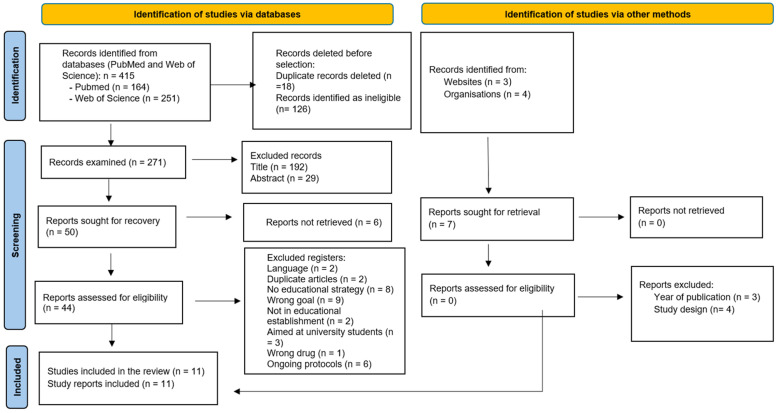
PRISMA 2020 flowchart for a scoping review based on database searches and other sources [31].

In the end, 11 articles were left for final analysis. The PRISMA flowchart of the study selection process is shown in Figure 1.

### 3.2. Characteristics of the Items Included

Among the selected items’ characteristics, the following properties stand out. In terms of study design, eight studies (72.8%) used a randomised controlled method, one study (9.1%) used a pre-experimental design with pretest and follow-up evaluation, and one study (9.1%) was defined as an implementation trial (hybrid implementation-effectiveness design), and 1 (9.1%) used the mixed methods feasibility trial method.

Regarding the age of the target population, 2 (18.2%) studies focused on school children aged 15–19 years, 1 (9.1%) on school children aged 11–14 years, and 1 (9.1%) on students aged 12–13 years. In addition, 1 (9.1%) study targeted school children aged 12–14 years, 1 (9.1%) targeted student aged 12–15 years, 1 (9.1%) targeted schoolchild aged 13–14 years, 1 (9.1%) targeted child aged 14–16 years and 1 (9.1%) targeted child aged 14–17 years. 2 articles did not define the age range of the target population, but mentioned the mean age, which was 14.1 years in 1 (9.1%) article and 17.3 years in another (9.1%) study.

In terms of the geographical distribution of the studies, there is a remarkable heterogeneity. Of the 1 study conducted on the American continent, 1 (9.1%) was conducted in the United States. In Europe, 2 (18.2%) studies were conducted in Switzerland, 1 (9.1%) in Spain, 1 (9.1%) in the UK and 1 (9.1%) in Poland. There was also 1 (9.1%) study in Africa, specifically in South Africa, and 1 (9.1%) in Asia, originating from China. Finally, 3 (27.2%) studies were conducted in Oceania, specifically in Australia.

A summary of these findings is presented in Table 1.

The results of the articles were categorised into three topics: (1) Design and Focus of Educational Strategies; (2) Implementation Barriers and Enablers; (3) Absence of School Nurses and Policy Implications (see Table 2 below for a detailed summary).

**Design and Focus on Educational Strategies**

In the selected articles, various educational strategies implemented in schools to reduce alcohol and tobacco use among school-age children were identified. All strategies evaluated the reduction of consumption after implementation. These included fully online (45.5%), hybrid (27.3%), and face-to-face (27.3%) formats.

Most strategies targeted alcohol alone (36.4%) or in combination with tobacco or cannabis [36,41,45]. Some interventions also addressed other substances such as cannabis, methylenedioxymethamphetamine (MDMA), and prescription drugs [35,37,46].

The objectives of these programs varied: over half (54.5%) aimed to develop social and self-regulation skills [34,35,36,42], while others focused on harm reduction [46], awareness through information [37], or building resilience and coping strategies [39,40]. One study also addressed mental health [41].

Methodologies included:

- Mobile-based interventions (Ready4Life, SmartCoach) [35,37].
- Interactive storytelling and computer-tailored feedback (Alcohol Alert, Climate Schools) [40,41].
- Debates and role-play (HealthWise, Debate program) [36,39]
- Online games and quizzes [38].

Several programs were grounded in theoretical models such as social cognitive theory, the I-Change model, and the IMB model, reflecting an emphasis on behavioural change rather than purely informational approaches.

In terms of outcomes, two studies showed significant reductions in both [34,46], while others reported reductions in either alcohol [35] or tobacco [36,37]. Some interventions showed non-significant changes [41,45], and one showed no change [42].

**Implementation Barriers and Enablers**

Several studies highlighted the feasibility and cost-effectiveness of school-based educational strategies (63.6%) [34,35,37,38,39,41]. These benefits were often linked to the methodology and delivery format, particularly online and hybrid models. Flexibility was also noted as an advantage in two studies: one emphasised the adaptability of the IMB model [42], and another highlighted the accessibility of online formats [45]. Additionally, preserving core components during digital delivery [46] and involving teachers in implementation [36] were seen as strengths.

However, several limitations were reported. Low participation by students and parents in online activities was observed in four studies (36.4%) [35,38,45], and one study reported that some content was perceived as long and unengaging [40]. These issues were particularly salient in digital and hybrid interventions, where the absence of structured follow-up and monitoring mechanisms often led to reduced participation and inconsistent delivery.

Implementation fidelity was another concern, with four studies (36.4%) reporting that teachers made modifications affecting program delivery [34,36,42,46]. The lack of institutional support and professional development for teachers was evident in studies such as [34,45], in which educators were responsible for implementation without formal training or coordination mechanisms. Other limitations included the declining long-term effectiveness of digital interventions [37], potential bias in evaluation tools [39], and the need for longer follow-up periods to assess sustained impact [41].

**Absence of School Nurses and Policy Implications**

None of the 11 studies (0%) included in this review explicitly mentioned the involvement of school nurses in the design, implementation, or evaluation of educational strategies to prevent alcohol and/or tobacco use in school settings. All interventions were delivered by teachers or external facilitators, often without institutional support or interprofessional collaboration, and with no reference to nursing professionals in the school context. Only one study [36] mentioned the relational role of teachers, but did not involve healthcare professionals.

Although some grey literature sources referenced nurses’ roles in broader health promotion, these documents were excluded because they did not meet the study’s eligibility criteria. As a result, no empirical evidence was found on the participation of school nurses in the analysed interventions.

Furthermore, none of the included studies addressed policy frameworks, institutional guidelines, or recommendations on integrating school nurses into substance use prevention programs. This absence was consistent across all studies, regardless of country, intervention format, or target population.

## 4. Discussion

This scoping review mapped recent school-based educational strategies aimed at preventing alcohol and tobacco use among adolescents. The findings reveal emerging trends in programme design and delivery, particularly the growing reliance on digital and hybrid formats, alongside persistent challenges related to student engagement and implementation fidelity. Within this landscape, the absence of school nurses in programme design, delivery, or evaluation is a notable gap, especially given their well-established role in school health promotion.

**Intervention Strategies and Emerging Patterns**

The most commonly identified interventions were digital or hybrid, reflecting global trends toward scalable, technology-enhanced solutions in school health promotion [34,35,45,46]. These approaches often incorporate interactive storytelling, mobile applications, and online modules to foster social and self-regulation skills [43]. However, their effectiveness appears nuanced: while some studies reported short-term reductions in alcohol and tobacco use, long-term outcomes remain uncertain [37,38,39].

This variability is consistent with previous reviews showing that technology-based programmes can expand accessibility but often struggle to sustain behavioural change without ongoing reinforcement [44,47]. Our findings echo this pattern: interventions relying solely on digital delivery tended to show short-term improvements but lacked mechanisms to maintain engagement over time. Similarly, initial benefits often diminish without continued support, suggesting that accessibility alone is insufficient to ensure lasting impact [9]. In contrast, hybrid interventions, combining online content with face-to-face components, have been highlighted as more promising, as they appear better equipped to foster relational engagement, contextual adaptation and opportunities for guided reflection [48]. These comparisons suggest that the delivery format may shape not only reach but also the depth and durability of behavioural change, reinforcing the need for models that integrate digital efficiency with interpersonal support.

Although most interventions identified in this review incorporated digital or hybrid components, only a subset included active, participatory or skill-building strategies, elements consistently associated with stronger and more sustained behavioural outcomes [49]. This pattern mirrors findings from previous reviews, which emphasise that digital delivery alone rarely produces lasting change unless it is paired with opportunities for practice, reflection and interpersonal interaction [50]. Health-promotion frameworks offer a useful lens for interpreting these results. Social Cognitive Theory highlights the importance of modelling, guided practice and self-efficacy [51]; Positive Youth Development approaches stress the need to strengthen personal competencies through experiential activities [52]; and the Developmental Assets Framework underscores the role of protective factors embedded in supportive school and community environments [53].

Seen through these lenses, the limited use of structured skill-building activities in several interventions may help explain the variability in outcomes observed across studies. The broader literature suggests that effective prevention schools should prioritise social skills, self-regulation, decision-making and meaningful connections with adults [54]. Yet, these elements appeared inconsistently across the programmes included in this review.

**Implementation Challenges**

Several authors described these interventions as feasible and low cost, particularly for digital formats that required little more than internet access and basic devices [34,35]. However, these benefits were often offset by important challenges. Several studies reported low student and parental engagement, especially in online programmes that lacked relational support or follow-up mechanisms [38,45]. Issues of implementation fidelity also emerged when interventions were delivered solely by teachers, who sometimes modified or skipped sessions [36,38,46].

Across the included studies, authors noted that although digital and hybrid interventions were often described as feasible and low cost, several studies reported important barriers during real-world implementation. Although these programmes were easy to deploy, several struggled to sustain participation, particularly in online formats without relational support or structured follow-up [38,45]. This helps explain why interventions that appeared highly scalable did not always achieve meaningful or lasting engagement. These findings align with previous research showing that digital approaches often lose momentum over time when they rely on passive content or offer limited opportunities for interaction [55]. In line with broader school-based health promotion literature, relational elements, such as teacher, student rapport, peer interaction and guided reflection, remain central for engagement, and their absence can weaken programme uptake [56].

Implementation fidelity also emerged as a key challenge. In several studies, when teachers were solely responsible for delivering the programmes, sessions were modified, condensed or skipped altogether [36,38,46]. This aligns with previous research showing that teacher-led interventions without external support are particularly prone to drift, inconsistency and reduced impact [57]. Competing demands, limited time and insufficient training have also been identified as barriers that make it difficult for teachers to follow structured protocols as intended [58]. These constraints may help explain the variability in programme outcomes, as inconsistent delivery can dilute core components and reduce the likelihood of meaningful behavioural change.

Family engagement was another recurring challenge. Several studies reported difficulties in involving parents, particularly in digital formats where communication tended to be limited or optional [38,45]. This is consistent with evidence showing that parental participation is one of the most difficult aspects of school health initiatives, even though it plays a key role in reinforcing messages and supporting behaviour change at home [59]. Reviews on children substance-use prevention also highlight that programmes without meaningful family involvement often show weaker or less durable effects, suggesting that the lack of parental reinforcement may undermine long-term outcomes [60].

Addressing these challenges may require more structured implementation support, clearer guidance for educators and ongoing professional development to ensure fidelity in programme delivery [61]. Several authors also highlight the value of implementation frameworks that include coaching, monitoring and feedback loops, which can strengthen fidelity and sustain engagement. Reinforcing relational elements, such as dialogue, mentoring and group interaction, seems especially important for digital interventions that risk becoming passive or disconnected from students’ experiences [62]. Greater collaboration among teachers, families and school health professionals could further enhance consistency and engagement, helping to close the gap between theoretical feasibility and real-world effectiveness [63].

**Absence of Nursing Professionals**

A notable finding of this review was the complete absence of school nurses in the design, delivery or evaluation of the interventions, despite policy frameworks that explicitly promote whole-school collaboration [64,65]. This gap is striking given the well-established role of school nurses in adolescent health: they are uniquely positioned to bridge education and healthcare systems, provide continuity of care, and identify risk behaviours at an early stage [66,67].

Evidence from other contexts reinforces this potential. For instance, nurse involvement in alternative school settings has been shown to strengthen substance-use screening and referral pathways [68]. In contrast, recent work on adolescent mental health highlights how nurses can enhance programme responsiveness and support more consistent implementation [69]. Their absence in the studies reviewed may therefore reflect broader structural barriers rather than a lack of relevance. Schools often operate under strong academic pressures, limited resources and fragmented governance structures, which can marginalise health professionals and hinder the integration of health expertise into educational initiatives [70].

This disconnect perpetuates a siloed model in which educational and health objectives run in parallel rather than reinforcing one another, despite evidence that addressing risk behaviours can improve academic engagement and overall school functioning [71]. The omission of nursing professionals may thus limit the depth, continuity and relational quality of prevention efforts, particularly in programmes that rely heavily on teachers or digital formats.

**Implications for Policy and Research**

This review fills an important gap by mapping recent school-based strategies to prevent alcohol and tobacco use among adolescents and by highlighting the complete absence of school nurses in these interventions. From a policy perspective, the findings underscore the need for frameworks that explicitly integrate health professionals into school prevention efforts, as their involvement could help address persistent barriers such as low engagement and inconsistent delivery.

For practice, the synthesis offers actionable insights into the strengths and limitations of current delivery formats—digital, hybrid and face-to-face—and the contextual factors that shape their feasibility. Schools and education authorities can draw on this evidence to select approaches that align with their resources and to plan mechanisms to strengthen fidelity and participation.

For research, two priorities emerge: evaluating models that incorporate school nurses into prevention programmes, and testing strategies that enhance engagement and long-term effectiveness in technology-based interventions. The findings also point to the value of broader reviews that include integrated health-promotion initiatives, even when substance use is not the primary focus.

These implications provide a roadmap for developing prevention efforts that are more effective, sustainable and sensitive to the realities of school settings.

### Strengths and Limitations

This scoping review has several limitations. Search was restricted to two databases (PubMed and Web of Science) and a five-year window, which may have excluded relevant studies. The review was also limited to publications in English and Spanish, which may have excluded relevant studies available in other languages. In addition, the strategy focused on interventions explicitly addressing alcohol and tobacco use in school settings, potentially overlooking programs led by school nurses or broader health promotion initiatives where these substances were not mentioned. Moreover, the nursing-related search component was exploratory rather than fully integrated into the primary search strategy, and this should be considered a limitation of the review. These constraints limit the scope of the findings and highlight the need for future reviews with broader search strategies and inclusion criteria.

Despite these limitations, the review has important strengths. It adhered to PRIS-MA-ScR and JBI guidelines, ensuring methodological rigour and transparency. A collaborative and iterative approach to screening and data extraction strengthened reliability, and the focus on recent evidence enabled an up-to-date synthesis aligned with current technological and policy trends. Furthermore, the review mapped diverse interventions across multiple countries and delivery formats, identified key implementation challenges, and documented the absence of nursing involvement, providing actionable insights for research, practice, and policy.

## 5. Conclusions

This scoping review shows that schools are implementing a variety of educational strategies to prevent alcohol and tobacco use among adolescents, with digital, hybrid and face-to-face formats each presenting specific advantages and challenges. Across the included studies, difficulties with engagement, follow-up and implementation fidelity were frequently reported, highlighting the need for support mechanisms that strengthen programme delivery in real school settings.

None of the interventions described in the included studies involved school nurses. While their participation is recognised in broader school health frameworks, its potential contribution should be understood as a contextual implication rather than as a finding derived from the evidence reviewed.

Overall, the results suggest that effective school-based prevention requires not only accessible formats but also relational support, consistent implementation and stronger collaboration within the school community. Future research could explore models that more systematically incorporate school nurses and examine approaches to improve engagement and long-term effectiveness, particularly in technology-based interventions.

## Figures and Tables

**Table 2:** Outcome of educational strategies, alcohol and/or tobacco use, benefits and limitations of educational strategies and the role of nursing.

Educational Strategies	N (%)
Use of mobile phones (through online comments, text messaging and recreational functions) to address substance use and promote social skills.	2 (18.2%)
Online stories (scenarios in which they were confronted with substance use), in which school children chose actions to develop the outcome of the story.	2 (18.2%)
Methodology of the debate.	2 (18.2%)
Role-play methodology	2 (18.2%)
Strategy based on IMB model (Information, Motivation and Behavioural Skills Model).	1 (9.1%)
Strengths-based learning through a case study strategy.	1 (9.1%)
Methodology of online games.	1 (9.1%)
**Results of educational strategies in the reduction of alcohol and/or tobacco consumption**	
**Strategies that addressed alcohol and tobacco substances**	**5 (45.4%)**
Decrease in alcohol and tobacco consumption. Statistically significant results for both substances.	2 (18.2%)
Decrease in alcohol and tobacco consumption. Statistically significant results only for alcohol consumption.	1 (9.1%)
Statistically significant decrease in tobacco consumption, but not in alcohol consumption.	2 (18.2%)
**Strategies that addressed the substances alcohol and cannabis**	**2 (18.2%)**
There was a decrease in alcohol consumption, but these results were not significant	2 (18.2%)
**Strategies that exclusively addressed the substance alcohol**	**4 (36.4%)**
A decrease in alcohol consumption, with these results being significant	1 (9.1%)
There was a decrease in alcohol consumption, but these results were not significant	2 (18.2%)
There was no decrease in alcohol consumption, but no increase either	1 (9.1%)
**Benefits of educational strategies**	
Feasibility and cost-effectiveness of the strategy	7 (63.6%)
Flexibility of strategy	2 (18.2%)
Preservation of the intervention’s core resources	1 (9.1%)
Involvement of teachers in the implementation of the intervention	1 (9.1%)
**Limitations of educational strategies**	
Low participation in strategy-related activities by school children and parents.	4 (36.5%)
Lack of fidelity in the implementation of programs when teachers carry them out.	4 (36.5%)
Low effectiveness of long-term digital strategies	1 (9.1%)
Imprecision in the program’s evaluation instrument	1 (9.1%)
It did not report limitations of the education strategy	1 (91%)
**Role of the school nurse in educational strategies**	
The role of the school nurse and nursing in general in educational strategies was not mentioned or included in any of the articles.	0 (0%)

N (%): number of articles reported (percentage)—percentage calculated on the total number of articles included in the review (N = 11).

## Data Availability

Not applicable.

## Supplementary Materials

The following supporting information can be downloaded at: https://www.mdpi.com/article/10.3390/children13040453/s1, Supplementary Material S1: Preferred Reporting Items for Systematic reviews and Meta-Analyses extension for Scoping Reviews (PRISMA-ScR) Checklist; Supplementary Material S2: Main Search Strategy.

## Abbreviations

The following abbreviations are used in this manuscript:

AEPAP	Spanish Association of Primary Care Paediatrics
CDC	Centres for Disease Control and Prevention
CSP	Climate Schools Plus
EU	European Union
FCTC	Framework Convention on Tobacco Control
JBI	Joanna Briggs Institute
MeSH	Medical Subject Headings
NASN	National Association of School Nurses
PRISMA-ScR	Preferred Reporting Items for Systematic Reviews and Meta-Analyses Extension for Scoping Reviews
PPEE	Prevention Programme in Educational Establishments
RCT	Randomised Controlled Trial
SDG	Sustainable Development Goal
SPSS	Statistical Package for the Social Sciences
