# Supplementary material for: School-Based Alcohol and Tobacco Prevention Strategies: A Scoping Review and the Missing Role of School Nurses
Source: Children (Basel). 2026 Mar 26;13(4):453. doi: 10.3390/children13040453 (PMC13114386; doi:10.3390/children13040453)
Supplement: Supplementary file 1 [file children-13-00453-s001.zip › Supplementary material S2_Main Search Strategy.pdf]

Table S2. Main Search Strategy

Database	Consultation	Limiters and expanders	Last access made through	Results
PubMed	(("Adolescent"[MeSH Terms] OR adolescent*[Title/Abstract]) AND ("Schools"[MeSH Terms] OR school*[Title/Abstract]) AND (("Alcohol Drinking"[MeSH Terms] OR alcohol*[Title/Abstract]) OR ("Tobacco Use"[MeSH Terms] OR tobacco*[Title/Abstract])) AND (prevent*[Title/Abstract] OR "Prevention and Control"[MeSH Subheading]))	Publication date: 2019–2024; Languages: English, Spanish; Filters: Abstract, Classical Article, Clinical Study, Clinical Trial, Controlled Clinical Trial, Observational Study, Randomized Controlled Trial; Exclude preprints; Age: Adolescent (13–18 years)	Advanced Search Database - PubMed	162
	Complementary search (exploratory)<br><br>("School Nursing"[MeSH Terms] OR nurse*[Title/Abstract]) AND ((excludepreprints[Filter]) AND (fha[Filter]) AND (classicalarticle[Filter] OR clinicalstudy[Filter] OR clinicaltrial[Filter] OR controlledclinicaltrial[Filter] OR observationalstudy[Filter] OR randomizedcontrolledtrial[Filter]) AND (english[Filter] OR spanish[Filter]) AND (adolescent[Filter]) AND (2019:2024[pdat]))	Publication date: 2019–2024; Languages: English, Spanish; Filters: Abstract, Classical Article, Clinical Study, Clinical Trial, Controlled Clinical Trial, Observational Study, Randomized Controlled Trial; Exclude preprints; Age: Adolescent (13–18 years)		2
Web of Science	TS=(("school-based" OR "school health" OR "school program") AND adolescent*	Limiters – Release date: 5 years; Language: English,	Interface – WOS Core Collection	245

	AND (alcohol* OR tobacco*) AND (prevent* OR "educational intervention" OR "health education")) NOT (college OR university)	Document Type (Article), Languages (English OR Spanish), Timespan (2019–2024), Indexes (Core Collection).	
	Complementary search (exploratory)<br><br>(("school-based" OR "school health" OR "school program") AND adolescent* AND (alcohol* OR tobacco*) AND (prevent* OR "educational intervention" OR "health education")) NOT (college OR university)	Document Type (Article), Languages (English OR Spanish), Timespan (2019–2024), Indexes (Core Collection).	6

Note: The search strategies were developed based on the PCC framework (Population, Concept, Context) and adapted to each database. Filters applied included publication date (2019–2024), language (English and Spanish), and study type (empirical research). Complementary searches using nursing-related terms were exploratory and yielded no eligible studies. Full strategies are provided to ensure reproducibility.
